# Supplementary material for: TAR syndrome causal gene RBM8A is critical for embryonic bone development and proper Hedgehog signaling
Source: bioRxiv. 2026 Apr 23:2026.04.21.718480. Preprint. [Version 1] doi: 10.64898/2026.04.21.718480 (PMC13131530; doi:10.64898/2026.04.21.718480)

Supplementary Figure 1

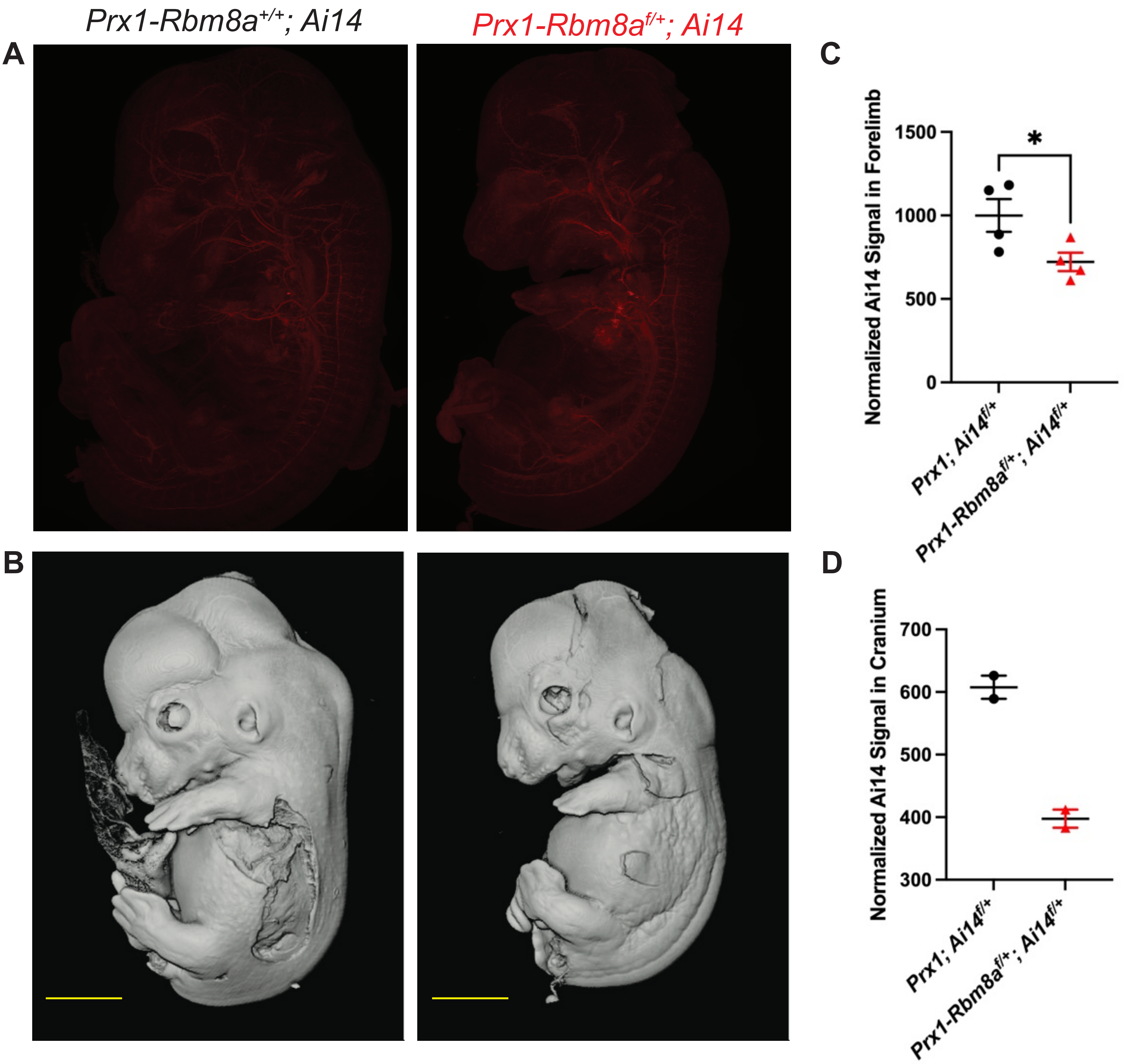

Supplementary Figure 2

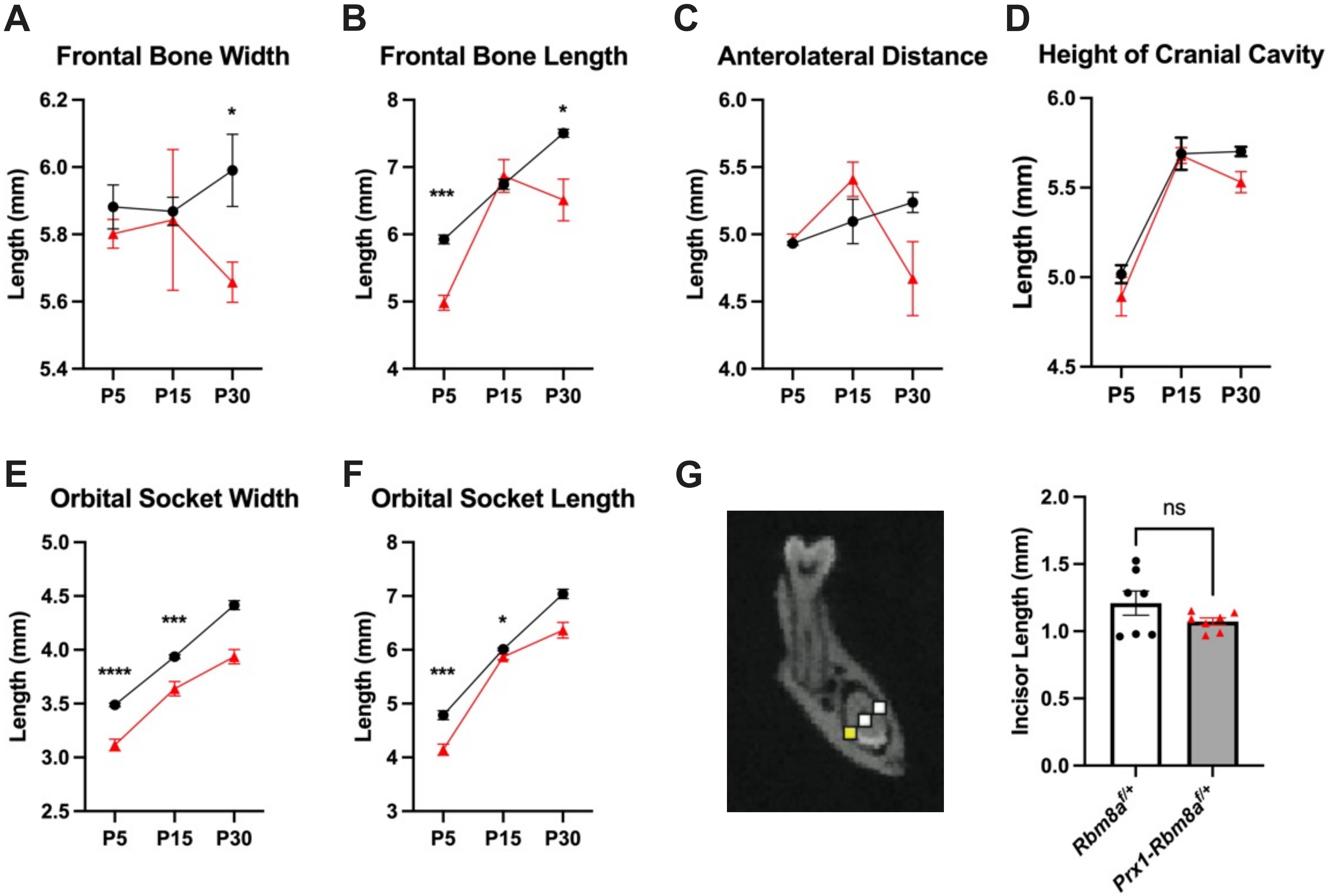

Supplementary Figure 3

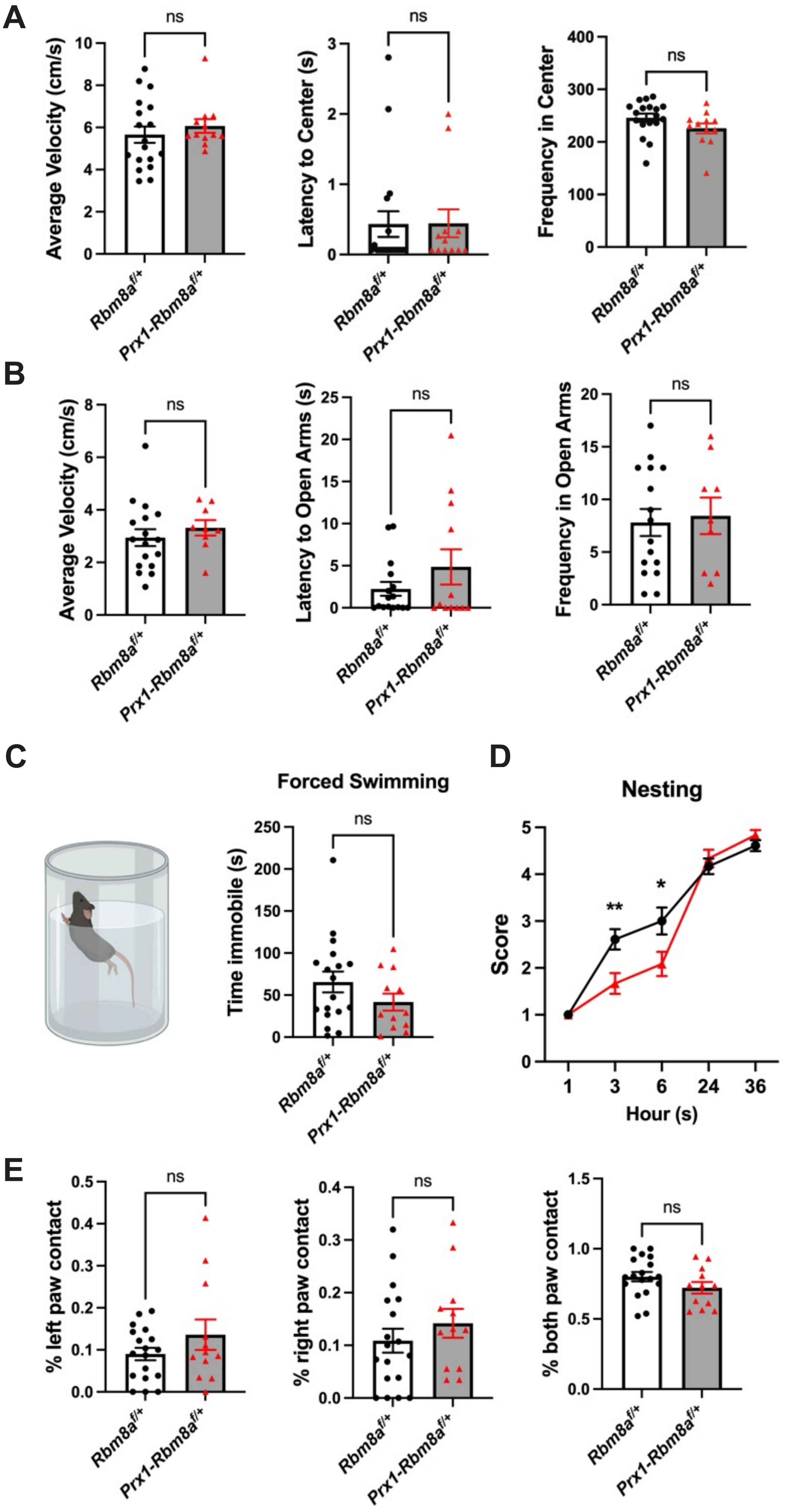

Supplementary Figure 4

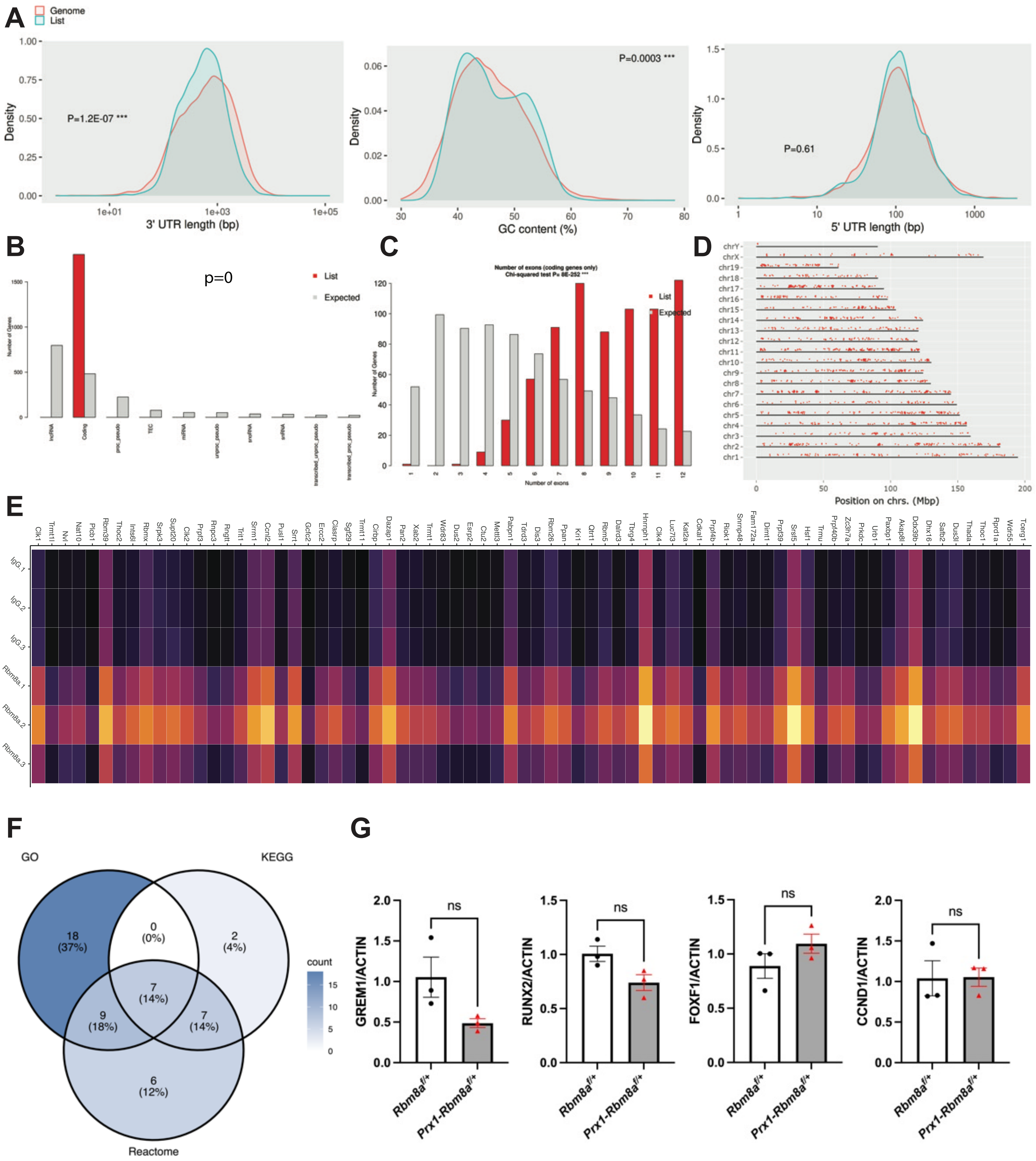

Supplementary Figure 5

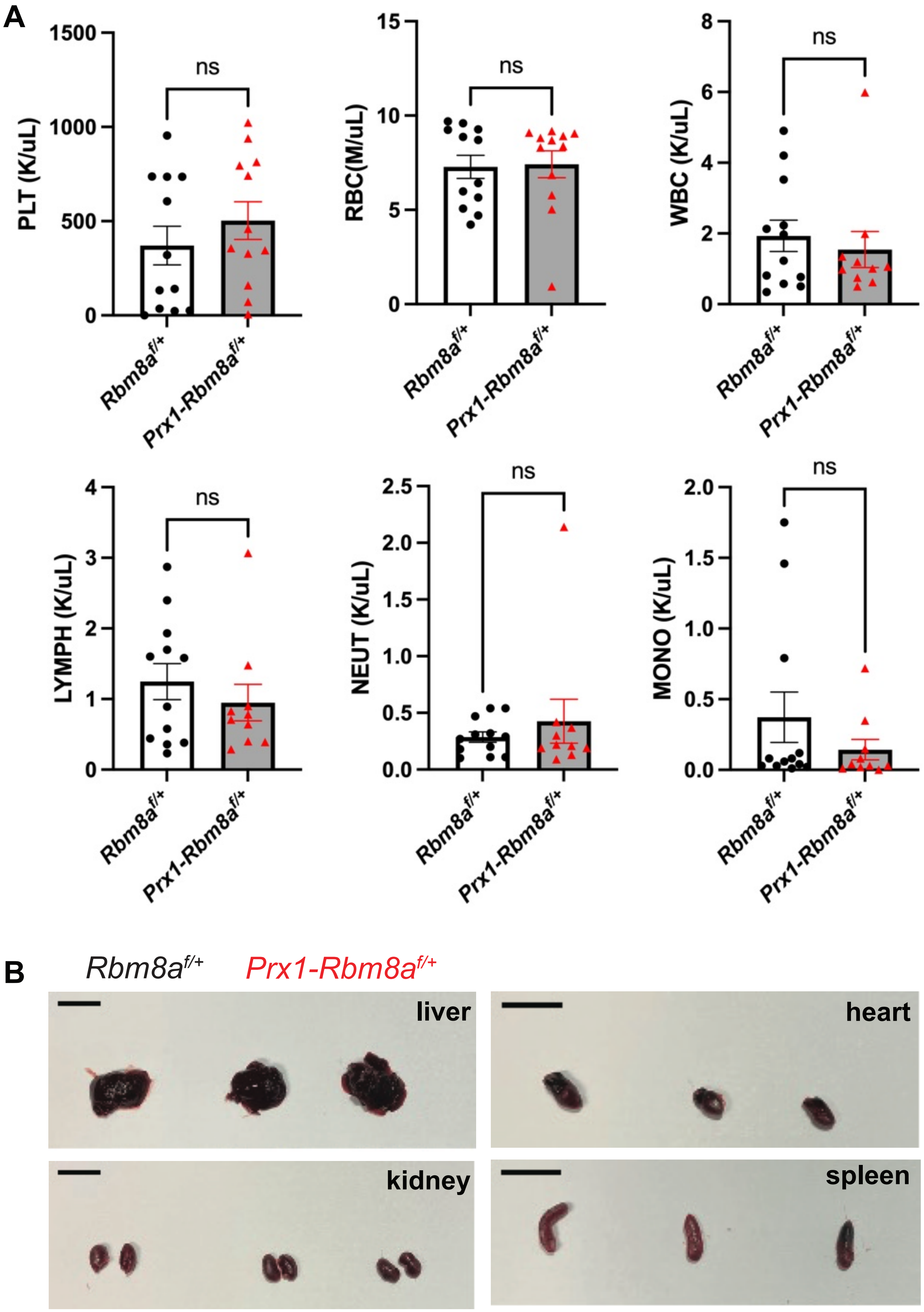

Supplementary Figure 6

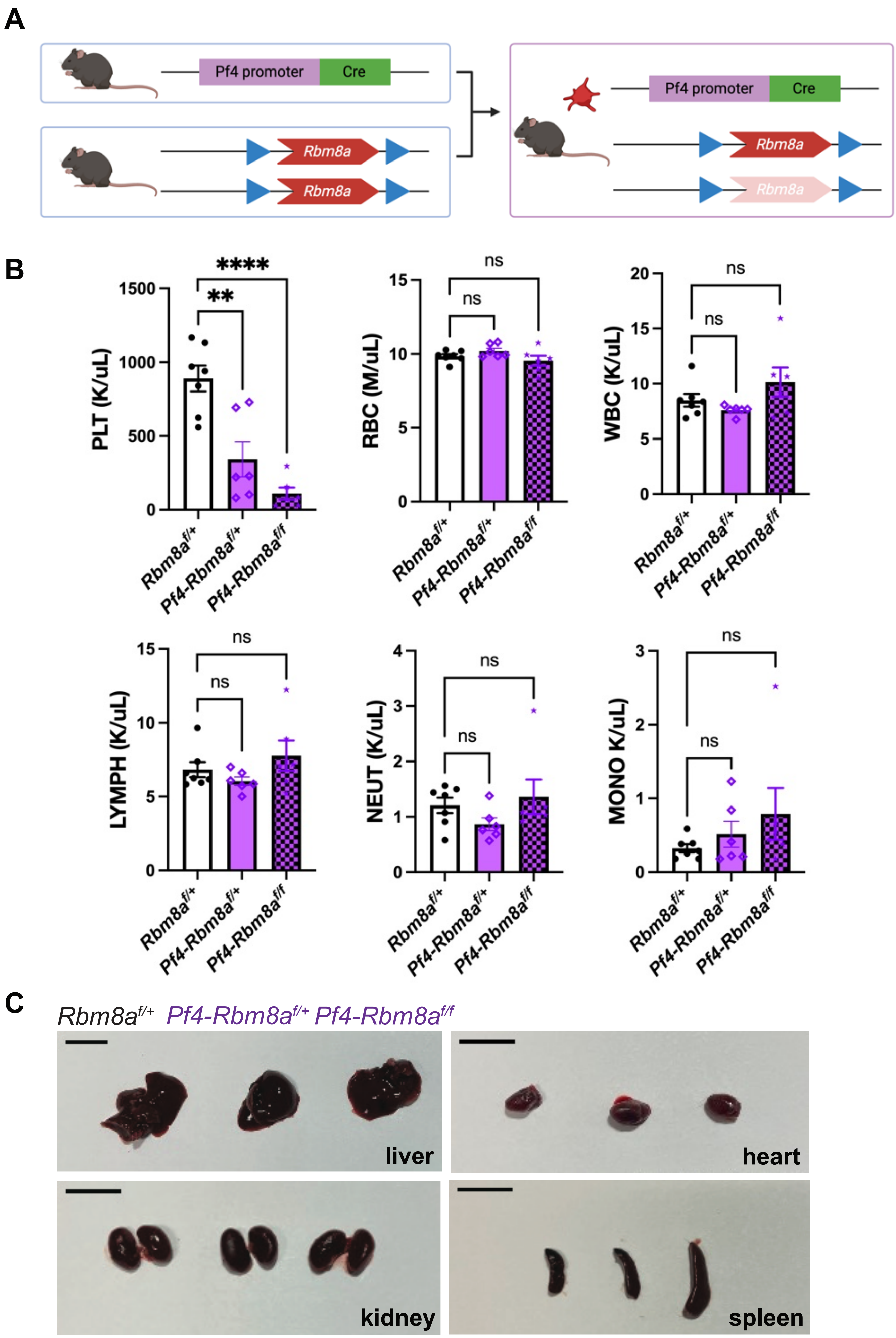

Supplement: Supplement 1 — Figure S1: Tissue optical cleared E15 Prx1; Ai14f/+ mice (control) and Prx1-Rbm8af/+; Ai14f/+ embryos. A. Lightsheet imaging of tissue optical cleared E15 Prx1; Ai14f/+ (control) and Prx1-Rbm8af/+; Ai14f/+ embryos showing Ai14 (tdTomato) reporter signal. B. Representative 3D rendering images of tissue optical cleared E15 Prx1; Ai14f/+ (control) and Prx1-Rbm8af/+; Ai14f/+ embryos. Scale bar = 100 μm. C. Quantification and statistical analysis of area-normalized Ai14 signal intensity in the forelimbs of E15 Prx1; Ai14f/+ (control) and Prx1-Rbm8af/+; Ai14f/+ embryos. Ai14 signal intensity in the forelimbs was significantly reduced in Prx1-Rbm8af/+; Ai14f/+ embryos compared to controls. Mean ± SEM (n=4 per group). Unpaired t-test. **, p < 0.01. D. Quantification and statistical analysis of area-normalized Ai14 signal intensity in the crania of E15 Prx1; Ai14f/+ (control) and Prx1-Rbm8af/+; Ai14f/+ embryos. Ai14 signal intensity in the crania was noticeably reduced in Prx1-Rbm8af/+; Ai14f/+ embryos compared to controls. Mean ± SEM (n=2 per group). Figure S2: Additional quantification of craniofacial landmarks of Prx1-Rbm8af/+ and control mice across developmental timepoints. Quantification and statistical analyses of (A) frontal bone widths, (B) frontal bone lengths, (C) distance between the anterolateral corners of the frontal bone, (D) height of cranial cavity between bregma and intersphenoidal synchondrosis, (E) orbital socket width, (F) orbital socket length, and (G) representative micro-CT scan image of incisor length (left) and quantification and statistical analyses (right) of incisor length of Prx1-Rbm8af/+ mice (red) and control mice (black) across developmental timepoints (P5, P15, P30). Mean ± SEM (n=3 per group). Unpaired t-test. *, p < 0.05, **, p < 0.01, ***, p < 0.001, ****, p < 0.0001. Figure S3: Additional results from behavioral tests on Prx1-Rbm8af/+ and control mice. A. Open field test results. Prx1-Rbm8af/+ mice and control mice had comparable averag [file media-1.pdf]
